# A recurrent mitochondrial p.Trp22Arg *NDUFB3* variant causes a distinctive facial appearance, short stature and a mild biochemical and clinical phenotype

**DOI:** 10.1136/jmedgenet-2015-103576

**Published:** 2016-04-18

**Authors:** Charlotte L Alston, Caoimhe Howard, Monika Oláhová, Steven A Hardy, Langping He, Philip G Murray, Siobhan O'Sullivan, Gary Doherty, Julian P H Shield, Iain P Hargreaves, Ardeshir A Monavari, Ina Knerr, Peter McCarthy, Andrew A M Morris, David R Thorburn, Holger Prokisch, Peter E Clayton, Robert McFarland, Joanne Hughes, Ellen Crushell, Robert W Taylor

**Affiliations:** 1 Wellcome Trust Centre for Mitochondrial Research, Institute of Neuroscience, Newcastle University, Newcastle upon Tyne, UK; 2 National Centre for Inherited Metabolic Disorders, Temple Street Children's University Hospital, Dublin, Ireland; 3 Centre for Paediatrics and Child Health, Institute of Human Development, Faculty of Medical & Human Sciences, University of Manchester, & Manchester Academic Health Science Centre, Manchester, UK; 4 Department of Metabolic Paediatrics, Royal Hospital for Sick Children, Belfast, UK; 5 University of Bristol and Bristol Royal Hospital for Children, Bristol, UK; 6 Neurometabolic Unit, National Hospital for Neurology and Neurosurgery, London, UK; 7 Manchester Centre for Genomic Medicine, Central Manchester University Hospitals NHS Foundation Trust, Manchester Academic Health Science Centre, Manchester, UK; 8 Department of Paediatrics, The Royal Children's Hospital, Murdoch Children's Research Institute, University of Melbourne, Parkville, Australia; 9 Institute of Human Genetics, Helmholtz Zentrum München, German Research Center for Environmental Health, Munich, Germany

**Keywords:** mitochondrial disease, complex I deficiency, prognosis, dysmorphic features

## Abstract

**Background:**

Isolated Complex I deficiency is the most common paediatric mitochondrial disease presentation, associated with poor prognosis and high mortality. Complex I comprises 44 structural subunits with at least 10 ancillary proteins; mutations in 29 of these have so far been associated with mitochondrial disease but there are limited genotype-phenotype correlations to guide clinicians to the correct genetic diagnosis.

**Methods:**

Patients were analysed by whole-exome sequencing, targeted capture or candidate gene sequencing. Clinical phenotyping of affected individuals was performed.

**Results:**

We identified a cohort of 10 patients from 8 families (7 families are of unrelated Irish ancestry) all of whom have short stature (<9th centile) and similar facial features including a prominent forehead, smooth philtrum and deep-set eyes associated with a recurrent homozygous c.64T>C, p.Trp22Arg *NDUFB3* variant. Two sibs presented with primary short stature without obvious metabolic dysfunction. Analysis of skeletal muscle from three patients confirmed a defect in Complex I assembly.

**Conclusions:**

Our report highlights that the long-term prognosis related to the p.Trp22Arg *NDUFB3* mutation can be good, even for some patients presenting in acute metabolic crisis with evidence of an isolated Complex I deficiency in muscle. Recognition of the distinctive facial features—particularly when associated with markers of mitochondrial dysfunction and/or Irish ancestry—should suggest screening for the p.Trp22Arg *NDUFB3* mutation to establish a genetic diagnosis, circumventing the requirement of muscle biopsy to direct genetic investigations.

## Introduction

Mitochondrial respiratory chain disease is a significant cause of human disease with a population prevalence of approximately 1 in 5000 in adults and children.^1^ Symptoms can manifest in the neonatal period but onset is often later in infancy, early childhood or even delayed to adulthood. Patients may present with disease affecting a single organ or have a multisystemic disorder typical of conditions such as Leigh syndrome. Approximately 70% of paediatric mitochondrial disease cases are caused by nuclear gene variants, while ∼30% harbour defects involving mitochondrially encoded (mtDNA) genes.^2^ ^3^ Conversely, mtDNA mutations more often underlie adult mitochondrial disease presentations.^4^ Beyond these prevalence statistics, the clinical and genetic heterogeneity results in a complex diagnostic pathway that usually relies on biochemical analysis of a muscle biopsy to direct genetic testing. Sanger sequencing of genes selected and prioritised according to clinical phenotype and biochemical results, as well as tissue biopsies, are being replaced by next-generation sequencing (NGS) strategies including candidate gene panels^5^ and whole-exome sequencing.^6^ ^7^

Investigation of isolated Complex I deficiency is particularly amenable to an NGS-based strategy given the number of genes implicated in its pathogenesis, with 44 structural subunits and at least 10 ancillary proteins required for enzyme assembly. It is the most common paediatric mitochondrial respiratory chain deficiency and mutations have been described in at least 29 genes to date,^8^ almost all being associated with a poor clinical course and bleak prognosis.^8^ Here we report the clinical and molecular genetic investigation of 10 patients from 8 unrelated families who all harbour an identical homozygous c.64T>C, p.Trp22Arg *NDUFB3* mutation, affecting a Complex I accessory subunit, previously reported in association with severe neurological presentations.^9^ ^10^ Most of our patients had considerably milder presentations despite harbouring the same variant. Recognition of mild dysmorphic facial features common to our initial patients prompted screening for the p.Trp22Arg *NDUFB3* variant in similar patients, leading to five further genetic diagnoses. This report demonstrates that the c.64T>C, p.Trp22Arg *NDUFB3* mutation can be associated with good long-term prognosis and that recognition of a cluster of physical characteristics may enable rapid diagnosis of *NDUFB3-*related mitochondrial disease, circumventing invasive procedures or extensive genetic testing.

## Subjects and methods

All patient samples were referred to the nationally commissioned ‘Highly Specialised Mitochondrial Diagnostic Laboratory’ in Newcastle upon Tyne for investigation of a putative mitochondrial defect. A clinical summary for each patient is given in table 1; detailed case reports are provided as online supplementary information. Informed parental consent was obtained.

**Table 1:** Clinical and biochemical findings in the patient cohort

							Physical appearance				
Patient (sex)	Ancestry	Clinical Presentation	Gestational age and birth weight (centile)	Age at latest review	Height at review (centile)	Lactate	Short stature	Prominent forehead	Long/thin philtrum	Residual CI activity*	Identified by
1 (M)	English	RSV+ acute respiratory collapse and hypoglycaemia aged 8 weeks requiring intubation for 8 days. Pulmonary hypertension on echocardiogram. Maximum-recorded lactate 14 mmol/L. Discharged after 18 days. Normal cardiac function and morphology at 13 months.	Term <0.4th	9.5 years	<0.4th	+++	+	+	+	35%	Targeted NGS panel.
2 (F)	Irish	IUGR. Acute life-threatening event, age 20 days, required intubation. Hypertrophic cardiomyopathy.	30weeks 2nd	6 years	2nd	+	+	+	+	33%	Targeted NGS panel.
3 (F)	Irish	IUGR and oligohydramnios, FTT, mild hypertrophic cardiomyopathy.	34weeks 2nd–9th	3.5 years	0.4th–2nd	++	+	+	+	32%	Targeted NGS panel.
4 (F)	Irish	Growth restriction. Ketotic hypoglycaemia following vomiting illness. Short stature prompted endocrinology referral. Growth hormone therapy. MRI: high signal in periventricular white matter and dentate nuclei.	39weeks 0.4th–2nd	8 years	n.d.	++	+	+	+	24%	Mutation screen.
5 (M)	Irish	IUGR. Poor feeding. Congenital hypothyroidism (strong paternal family history). Developmental delay, growth failure, FTT, learning difficulties. Endocrinology review for short stature.	37weeks 0.4th–2nd	10 years	0.4th	+	+	+	+	35%	Mutation screen.
6 (F)	Irish	Oligohydramnios. IUGR. Poor feeding at birth. MRI brain and echocardiogram normal. Age-appropriate skills. Family history of previous neonatal death.	37weeks <0.4th	2.5 years	2nd–9th	+++	+	+	+	35%	Mutation screen.
7 (M)	Irish	Sib of P6. IUGR. Normal echocardiogram and cranial ultrasound. Normal development.	36weeks 2nd–9th	10 months	9th	++	+	+	+	n.d.	Mutation screen.
8 (M)	Irish	Initial poor feeding. Short stature prompted endocrinology review. Growth hormone therapy. MRI: high signal in globus pallidus. Echo: murmur. ECG: Wolff–Parkinson–White syndrome.	Term 0.4th–2nd	9.5 years	2nd	−	+	+	+	n.d.	Whole-exome sequencing; endocrinology.
9 (F)	Irish	Sib of P8. IUGR. Growth hormone therapy. Normal MRI brain, echocardiogram and ECG.	Term <0.4th	8 years	2nd	−	+	+	+	n.d.	Whole-exome sequencing; endocrinology.
10 (M)	Irish	IUGR, chronic lung disease, growth restriction and weight faltering. Dysmorphic with partial agenesis of corpus callosum. Acute collapse with rhinovirus bronchiolitis, severe pulmonary hypertension at 5.5 months. Elevated lactates with intercurrent illnesses.	31weeks <0.4th	11 months	<0.4th	+++	+	+	+	36%	Mutation screen.

*Residual Complex I activities, normalised to the activity of the matrix marker enzyme citrate synthase, are expressed as a percentage of mean control values. FTT, failure to thrive; IUGR, intrauterine growth restriction; N.D., not determined; NGS, next-generation sequencing; RSV, respiratory syncytial virus.

10.1136/jmedgenet-2015-103576.supp1 Supplementary data

### Histochemical and biochemical analyses

Enzymatic activities of individual mitochondrial respiratory chain complexes were determined in patient muscle biopsies as previously described.^11^

### Targeted next-generation sequencing

A custom 84.38 Kb Ampliseq panel was designed using the Ion Ampliseq Designer V.2.2.1 (http://www.ampliseq.com) to target 49 genes implicated in Complex I deficiency (see online supplementary table S1). To generate the barcoded Ampliseq target library using the Ion AmpliSeq Library Kit 2.0 and Ion Xpress Barcode Adapter 1–96 Kit, 40 ng patient DNA was used. Libraries were quantified using an Agilent 2100 Bioanalyser and pooled at 100 pM for emulsion PCR and enrichment using the Ion OneTouch2 and Enrichment system. Sequencing using the Ion PGM 200 Sequencing Kit was performed using 316 chips on an Ion PGM Sequencer, all according to the manufacturer's protocol. Torrent Suite V.4.2.1 was used to align reads against the human genome (hg19). The Variant Caller plugin was used to identify sequence variants that were annotated using wANNOVAR.^12^

10.1136/jmedgenet-2015-103576.supp2 Supplementary table

### Whole-exome sequencing

Targeted enrichment and sequencing was performed using 3 µg patient DNA. Enrichment was performed using the Illumina HiSeq Sure Select All Exon v5 Enrichment Kit, and sequencing was performed on an Illumina HiSeq 2500 sequencer, all as directed. Sequence data were mapped with BWA software to the human genome (hg19). Variants were called using GATK V.2.4.7 software and annotated using Ensembl V.72. Ensembl's ‘defined consequence hierarchically’ system retained the highest impacting gene variant. Filtering removed variants with ≤5× coverage, a minor allele frequency (MAF)>1%, those predicted to be non-functional, and those reported in dbSNP138 (unless seen in the Human Gene Mutation Database (HGMD)) or an in-house database (n=647 exomes).

### Mutation screening, confirmation and carrier testing

The c.64T>C, p.Trp22Arg *NDUFB3* sequence variant was screened and confirmed using M13-tagged amplicons and Sanger sequencing with BigDye V.3.1 kit (Life Technologies). Capillary electrophoresis was performed using an ABI3130xl. Familial screening for the c.64T>C, p.Trp22Arg *NDUFB3* sequence variant was undertaken using parental and sibling DNA samples where available and appropriate.

### Haplotype analysis

A putative founder effect was investigated by genotyping two proximal (D2S309 and D2S2214) and two distal (D2S116 and D2S2309), short tandem repeat (STR) markers flanking the *NDUFB3* gene. Corresponding PCR primers are listed on Ensembl. Mapping distance was calculated using MAP-O-MAT.^13^

### Western blotting and blue native polyacrylamide gel electrophoresis

Mitochondrial fractions from control and patient muscle were prepared for western blotting and blue native polyacrylamide gel electrophoresis (BN-PAGE) as described previously.^14^ Protein concentrations were determined with the Pierce bicinchoninic acid (BCA) Protein Assay Kit. Muscle protein extracts (100 μg) were loaded on Native PAGE 4–16% BisTris gels, electrophoretically separated in the first dimension before proteins were immobilised onto a polyvinylidene fluoride (PVDF) membrane (Immobilon-P, Millipore Corporation) and subjected to standard immunoblotting analysis of oxidative phosphorylation (OXPHOS) complexes using primary and horseradish peroxidise conjugated secondary antibodies as described.^14^ For western blotting, equal amounts of muscle protein (50 μg) were loaded on 12% gels and resolved by sodium dodecyl sulfate-polyacrylamide gel electrophoresis (SDS-PAGE), followed by wet transfer to PVDF membrane and subsequent immunodetection.

## Results

### Clinical findings

**Figure 1:**
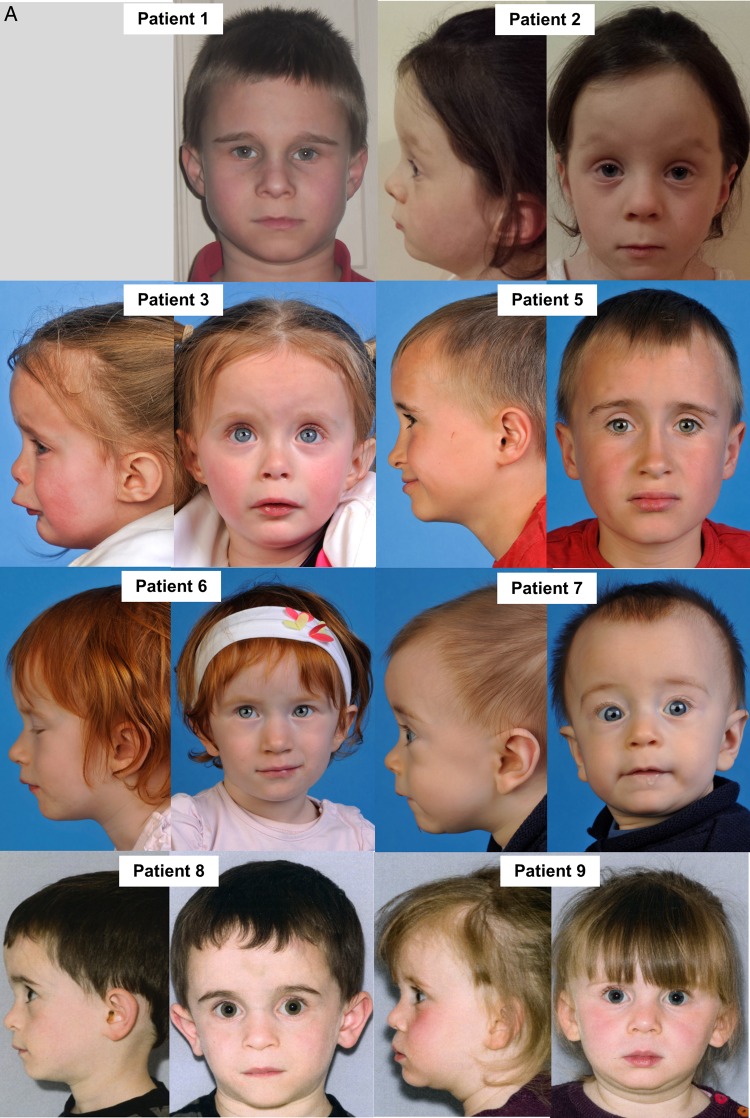

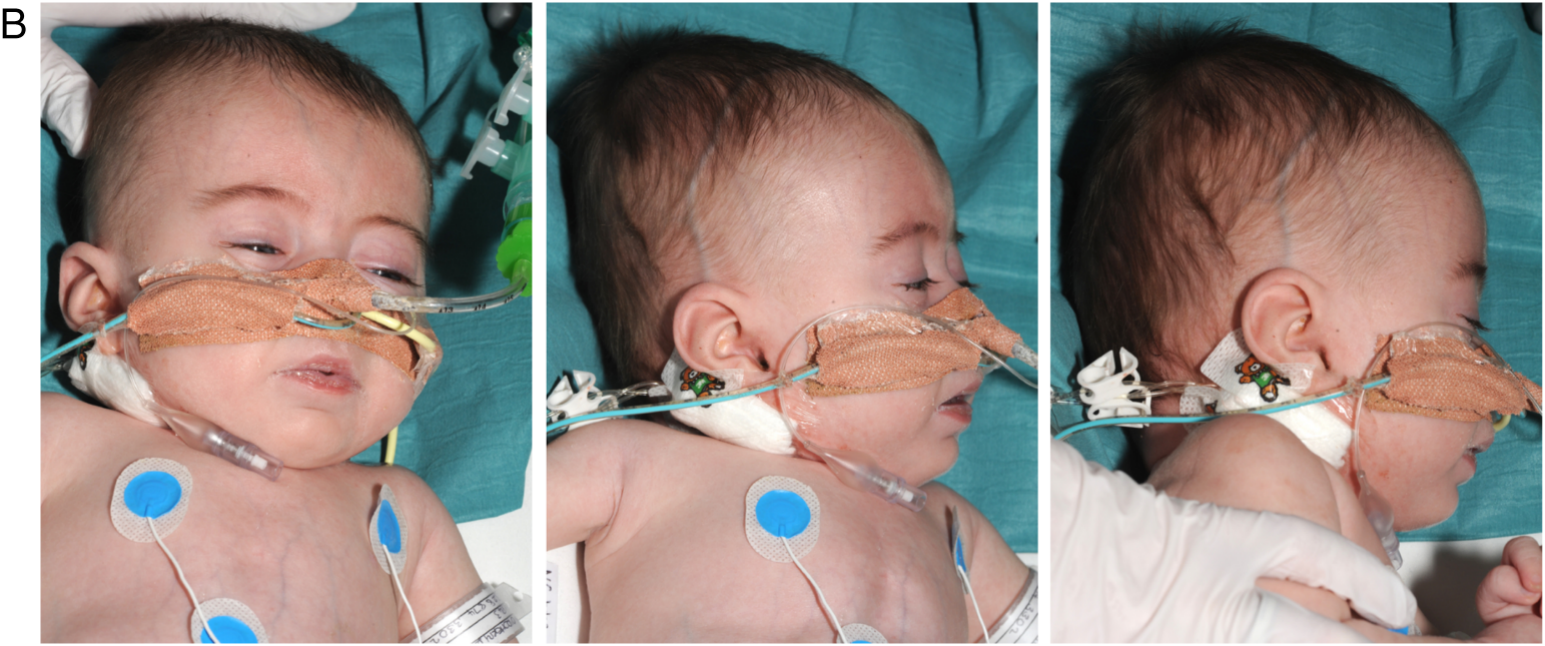
Clinical presentation associated with homozygous *NDUFB3* variant (A) Clinical photographs of eight patients harbouring a homozygous pathogenic c.64T>C, p.Trp22Arg *NDUFB3* variant. Patient 1 is of English descent, whereas the remaining cases are all of Irish heritage. Patients 6/7 and 8/9 are clinically affected sibling pairs. All have characteristic physical features including a prominent forehead, smooth philtrum, deep-set eyes and low-set ears. (B) Clinical photographs of patient 10, the youngest case within our cohort, illustrating the characteristic physical features associated with the p.Trp22Arg *NDUFB3* variant.

We describe five female and five male paediatric patients, each of whom are of short stature and share characteristic facial features. All weighed less than the 9th centile at birth, 8/10 were below the 2nd centile (80%). Clinical photography illustrates the prominent forehead, poorly defined philtrum and deep-set eyes (figure 1A, B). The majority of patients presented following a life-threatening metabolic crisis early in life followed by a period of sustained improvement. Subsequently, their clinical course has been largely benign but for occasional bouts of lactic acidosis associated with minor illnesses. A previous female sibling to patients 6 and 7 was born at term with growth restriction, became unwell and died on day 2 of life with profound lactic acidosis and multiorgan failure (no DNA was available for analysis). Patients 8 and 9 (siblings) presented to endocrinology for investigation of primary growth failure and were initially suspected to have 3M syndrome. Poor linear growth was seen in all patients, three patients have had growth hormone treatment with variable response.

### Histochemical and biochemical analyses of mitochondrial respiratory chain enzymes

Where muscle biopsy had been performed, we identified an isolated Complex I deficiency (table 1). No muscle biopsy was available for patients 8 and 9 as a metabolic condition was not suspected.

### Identification of a common underlying genetic defect

All patients in the cohort were found to harbour an identical homozygous c.64T>C, p.Trp22Arg *NDUFB3* sequence variant (table 1). Each of the three patients analysed by targeted NGS harboured between 54 and 57 genomic variants which were filtered to exclude those with a MAF >1% and variants outside the coding region ±10 bp of the intron/exon boundaries. For cases identified by whole exome sequencing (WES), from the 526 candidate variants compatible with autosomal recessive inheritance only a single, homozygous variant in *NDUFB3,* c.64T>C, p.Trp22Arg remained after filtering. All NGS-based strategies were confirmed by conventional Sanger sequencing.

The c.64T>C (chr2(hg38):g.201078946T>C) variant is referenced on dbSNP (rs142609245) and variant frequencies are recorded on ESP6500 (European: 14/8586 alleles (0.16%); African-American: 2/4404 alleles (0.05%)) and ExAC (Non-Finnish Europeans: 69/66 604 alleles (0.1%); African: 2/10 390 alleles (0.02%); Latino: 1/11 568 alleles (0.01%); South Asian: 9/16 484 alleles (0.05%)). There are no homozygous cases recorded on either ESP6500^15^ or ExAC^16^ databases. Although the highest prevalence is recorded in European populations, the presence of the c.64T>C, p.Trp22Arg *NDUFB3* variant in non-European populations suggests other independent occurrences of this pathogenic mutation.

### Carrier testing

With the exception of patients 4 and 5, where familial samples were unavailable, parental carrier testing confirmed recessive inheritance. Analysis of samples from the unaffected twin of patient 1 and the three unaffected siblings of patients 8 and 9 confirmed the homozygous genotype segregates with a clinically affected status.

### Haplotype analysis

Analysis of the *NDUFB3-*flanking STR markers across 0.5cM support multiple, independent occurrences of the c.64T>C, p.Trp22Arg variant (see online supplementary figure). Analysis of the markers most proximal to the *NDUFB3* gene (D2S309 and D2S2309), those most likely to be in linkage disequilibrium, shows three discrete haplotypes (1-1, 2-1 and 1-2). When including the distal STR markers in the analysis, this increases to seven haplotypes (*a*-*f*, plus #). There is one particularly prevalent haplotype (‘*a*’) in the patient cohort that is present in the heterozygous state in 8/10 cases and homozygous for 1/10 cases, supporting a founder allele. Additionally, the ‘*b’* and ‘*c*’ haplotypes are present in two unrelated families. Haplotype analysis of the two previously reported cases shows the variants are also on the background of either the ‘*a’* or ‘*b*’ haplotypes, suggesting a shared founder. We infer that the ‘#’ haplotype corresponds to the allele harbouring the truncating *NDUFB3* mutation reported by Haack *et al*, as patient RC1 harboured a p.Trp22Arg variant in compound heterozygosity with p.Gly70*.

10.1136/jmedgenet-2015-103576.supp3 Supplementary figure

### Steady-state levels of respiratory chain components and complexes

**Figure 2:**
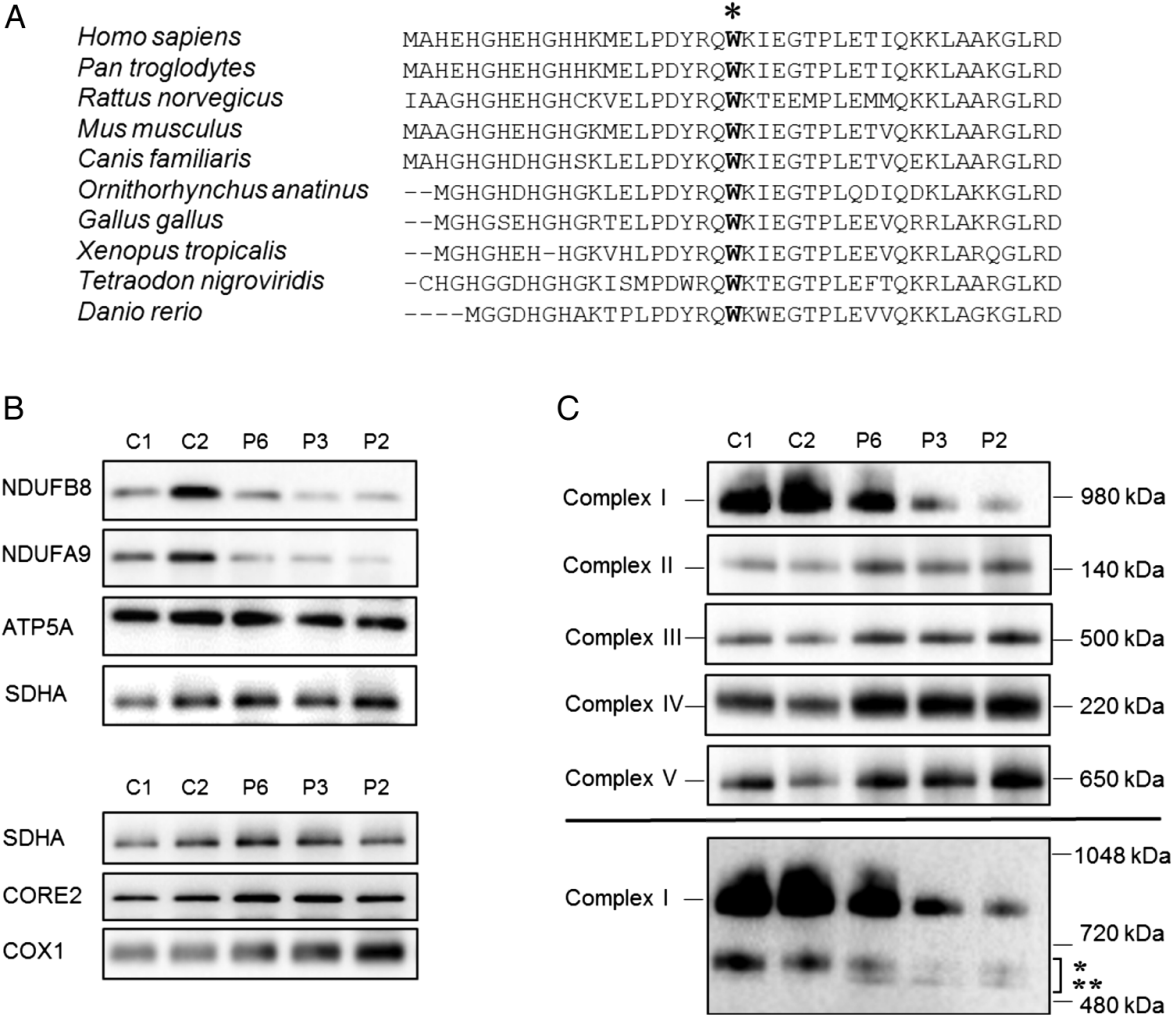
Analysis of OXPHOS complex assembly and protein expression levels (A) Clustal Omega sequence alignment shows the evolutionary conservation of the p.Trp22 residue (marked with asterix), based on the human sequence (amino acids 1–43). (B) Immunoblot analysis of steady state levels of OXPHOS subunits in mitochondrial lysates isolated from control (C1, C2) and patient skeletal muscle samples (P6, P3, P2). OXPHOS subunit-specific antibodies against the indicated proteins showed a marked decrease in Complex I subunits (NDUFB8 and NDUFA9) in patient samples compared with controls. (C) One-dimensional blue native polyacrylamide gel electrophoresis (PAGE) (4–16% gradient) analysis showing a defect in the assembly of Complex I in patients with the homozygous *NDUFB3* variant. Individual OXPHOS complexes were detected by immunoblotting using subunit-specific antibodies (Complex I (NDUFB8), Complex II (SDHA), Complex III (UQCRC2), Complex IV (COX1) and Complex V (ATP5A)). The assembly of Complexes II–V was normal in all three patient samples when compared with age-matched controls. The lower panel suggests a presence of additional, partially assembled Complex I intermediates in both control and patient samples; the upper band (indicated by *) is likely to represent the ∼650 kDa Iβ subcomplex of the hydrophobic membrane arm while the lower band (indicated by **) represents partially assembled intermediates which are only visible in patient samples. These were detected by probing with an antibody raised against NDUFB8 and are in agreement with published studies.^17^ In (B) and (C), SDHA (Complex II) was used as loading control.

The p.Trp22Arg variant affects an evolutionary conserved amino acid residue (figure 2A). We investigated the steady-state protein levels of OXPHOS subunits in muscle available from three patients harbouring a homozygous p.Trp22Arg *NDUFB3* variant by SDS-PAGE and immunoblotting. The steady-state levels of Complex I subunit proteins NDUFB8 and NDUFA9 were decreased in all three patients while levels of protein components of Complexes II, III, IV and V were normal (figure 2B). Analysis of the assembly of OXPHOS complex subunits into mitochondrial respiratory chain complexes was undertaken by BN-PAGE, showing a decrease of fully assembled Complex I in P6, P2 and P3 muscle—correlating with the recorded biochemical defect—while the assembly profile of Complexes II, III, IV and V were all normal (figure 2C). Immunoblotting with NDUFB8 appeared to show partially assembled Complex I intermediates of ∼650 kDa in patient muscle, consistent with other defects involving subcomplex Iβ of the hydrophobic membrane arm, of which NDUFB3 and NDUFB8 are both integral components.^17–19^

## Discussion

Mitochondrial disease presentations are frequently heterogeneous, with a paucity of genotype-phenotype correlations to direct molecular genetic testing even with a known biochemical diagnosis. We present a cohort of 10 patients from 8 non-consanguineous families who harbour a homozygous c.64T>C, p.Trp22Arg *NDUFB3* variant; together these patients represent a distinct clinical presentation. The majority of patients presented with intrauterine growth restriction (IUGR) and share characteristic facial features including a prominent forehead, smooth philtrum, deep-set eyes and low-set ears. All patients are short (height <9th centile) and while short stature is not uncommon in mitochondrial disorders, dysmorphic features are rare with the exception of *PUS1*^20^ and *FBXL4*^21^ mutations. *NDUFB3* encodes a structural Complex I subunit, and contrary to reported Complex I-deficient cases there were surprisingly few persistent features of mitochondrial disease; blood lactate levels were typically normal, although transient acidotic events were reported following illness leading, in some cases, to hospital admission before recovery. There were no seizures, ataxia or other neurological deficit noted; patients 2 and 3 had hypertrophic cardiomyopathy on echocardiography, but this resolved with time. All patients are reported to be well, with good levels of energy, attaining developmental milestones and making good progress at school (where appropriate). Patient 10 (<1 year of age) is much younger than the rest of our patient cohort, but is making excellent developmental progress (see online supplementary case reports).

With the exception of one patient (patient 1), all are reported to be of Irish ancestry. Interestingly, analysis of the *NDUFB3-*flanking STR markers supports multiple, independent occurrences of the c.64T>C, p.Trp22Arg variant, despite its prevalence in the Irish population. Across the 0.5cM region analysed, there are six different p.Trp22Arg alleles; given that this region is not a recognised recombination hot spot, it is likely that the mutation has arisen independently and recurrently although our data suggest a common founder for some cases and cannot fully exclude recombination as a contributory factor.

The c.64T>C, p.Trp22Arg *NDUFB3* variant is represented on the ExAC server (0/81/121214 (homozygous/heterozygous/alleles); MAF=6.6×10^−4^) and has been reported in the literature twice previously, once in compound with a nonsense mutation and once as a homozygote; functional complementation experiments confirmed *NDUFB3* as the causative gene defect in both cases.^9^ ^10^ The homozygous case reported by Calvo *et al*^9^ had IUGR (weight <3rd centile) and presented with hypotonia and lactic acidosis, required ventilation and died at 4 months of age. The other reported case was born at 35 weeks gestation with low birth weight (3rd centile), with severe lactic acidosis and ketosis developing by day 2. Despite an initially severe presentation, her symptoms ameliorated and she is reported to remain of short stature but suffers illness-induced bouts of lactic acidosis. An older sibling of patients 6 and 7 in our series died on day 2 of life with profound lactic acidosis and multiorgan failure. No underlying cause was identified but a metabolic disorder was suspected, prompting early metabolic investigation of subsequent siblings.

Functional investigation of available patient muscle biopsy revealed a marked decrease in steady state levels of Complex I structural subunits, and although BN-PAGE analysis showed fully assembled Complex I to be diminished, some fully assembled Complex I remains. This is in stark contrast to many Complex I structural subunit defects, where levels of fully assembled Complex I are often entirely depleted,^22^ and could provide an explanation for the milder clinical phenotypes observed in our patient cohort. Nuclear modifiers or mtDNA background could influence the prognosis but therapeutic intervention (see online supplementary case reports) and effective life support measures are most likely to contribute to the survival rate in our patients who presented in acute metabolic crisis. Evidence of mitochondrial proliferation was present in available muscle biopsy samples with elevated citrate synthase activity and ragged-red fibres indicating a cellular response to metabolic dysfunction. Our cohort and previously reported cases demonstrate this is a successful strategy for some but not all. Typically, paediatric mitochondrial disease patients progressively decline, with the exception of some patients with *TRMU* mutations or the m.14674T>C/G mt-tRNA^Glu^ ‘reversible cytochrome *c* oxidase (COX) deficiency’ variants. We show that the p.Trp22Arg *NDUFB3* mutation can also be associated with good long-term survival, even when some patients present in acute metabolic crisis with an isolated Complex I deficiency in muscle.

Another unique aspect of this case series involves patients 8 and 9; all other cases were referred by metabolic paediatricians but these children were diagnosed by paediatric endocrinologists investigating primary short stature. Blood lactates were normal for both children but patient 9 presented with Kussmaul-type respiration aged 2 years, which could be consistent with lactic acidosis. In light of the reported c.64T>C, p.Trp22Arg *NDUFB3* cases, cardiac screening was performed, revealing Wolff–Parkinson–White (WPW) syndrome in patient 8, a rare cardiac conduction defect which is over-represented in patients with mitochondrial disease.^23^ The initial manifestation of WPW syndrome can be sudden death and the diagnosis might facilitate interventions including non-invasive risk stratification and/or therapeutic ablation.^24^

Many cases of isolated Complex I deficiency associated with nuclear gene mutations are discrete entities and no common variant accounts for more than a few apparently unrelated cases.^25^ We present 10 patients from 8 families who harbour the same homozygous *NDUFB3* variant and share a plethora of unifying physical features, an unprecedented finding in association with isolated Complex I deficiency. Recognition of the distinctive facial features in combination with short stature should suggest screening for the c.64T>C, p.Trp22Arg *NDUFB3* mutation, even in the absence of ‘classic’ metabolic symptoms, and particularly when Irish ancestry is involved.
