# Supplementary material for: A recurrent mitochondrial p.Trp22Arg NDUFB3 variant causes a distinctive facial appearance, short stature and a mild biochemical and clinical phenotype
Source: J Med Genet. 2016 Apr 18;53(9):634–41. doi: 10.1136/jmedgenet-2015-103576 (PMC5013090; doi:10.1136/jmedgenet-2015-103576)
Supplement: Supplementary data [file jmedgenet-2015-103576supp.pdf]

Patient 1

This male is the fourth child of unrelated parents and was born at term weighing 2.2kg. He presented at 8 weeks old with a 4 week history of cough and poor feeding for 2 days. He experienced a respiratory collapse with profound acidosis (initial pH 7.1 falling to pH 6.9) and required artificial ventilation, nitric oxide and inotropic support within an intensive care unit. Although hypoglycaemia was not evident initially, his blood glucose fell subsequently to 1.2 mmol/L. Blood lactate measured 14.8mmol/L (normal <2.2mmol/l) with an ammonia level of 124. Co-enzyme Q10 (5mg/kg/day), riboflavin (12.5mg/kg/day), ascorbate 200mg and carnitine (100mg/kg/day) supplementation were commenced on day 2 of admission. Lactates improved (4.4mmol/L on day 2) and remained at 3-5mmol/L until day 13 when a level of 2.7mmol/L was recorded. He was discharged 18 days after admission and has had only minor health problems since then.

Normal laboratory investigations included creatinine kinase, acylcarnitine profile and organic acid screen. He had raised hydroxybutyrylcarnitine consistent with ketosis whilst amino acid profile showed elevated proline at 513µmol/L (normal: 40-332), alanine at 872µmol/L (normal: 120-600) and lysine 364µmol/L (normal: 66-270) consistent with lactic acidosis. Respiratory syncytial virus was isolated from naso-pharyngeal aspirate. INR was mildly deranged at 1.5, maximum ALT measured 303 (normal 5-40). Echocardiogram revealed pulmonary hypertension with a normal heart structure. A repeat echocardiogram at age thirteen months showed a normal heart. A muscle biopsy was taken for analysis of mitochondrial respiratory chain activities identifying low complex I activity. A glucagon stimulation test was performed aged five years to investigate his short stature that showed a normal growth hormone response.

At the age of 9.5years, he remains of low weight and height (20kg and 120.8 cm, respectively). Height velocity slowed to the 3rd centile between age 3 and 4 yr but has been subsequently maintained between 25th and 75th centiles. Weight has always been on or below the 0.4th centile. He has no education problems and is notably good at mathematics. He takes co-enzyme Q10 (20 mg/day) and riboflavin (50mg/day). There is no relevant family history with his three older siblings all clinically-unaffected.

Patient 2

This female dizygotic twin was born at 30+3/40 by emergency LSCS due to symmetrical intrauterine growth retardation, weighing 850g. Her clinically-unaffected twin sister weighed 1.79kg at birth. Initial course in the Neonatal Intensive Care Unit was uneventful until an acute life threatening event occurred on day 20 with profound metabolic acidosis (blood pH 6.7), requiring intubation and ventilation. Subsequent investigations revealed fasting blood lactate was 3.86mmol/L, rising to

5.4mmol/L post-feed (normal range 0.7-2.1 mmol/L); CSF lactate was 3.86mmol/L (normal <2.2mmol/L). LFTs and serum amino acids were normal. She had brief myoclonic jerks that were managed with phenobarbitone which was subsequently weaned in infancy. MRI brain was normal at this time, with a minor abnormality on EEG. Lactic acidosis (3 mmol/L) persisted with an elevated blood lactate:pyruvate ratio of 29. Urine organic acid analyses showed persistent increased excretion of lactate and TCA intermediates. CoEnzyme Q_{10} (dose 5mg/kg/day) vitamin C, vitamin E and riboflavin supplementation was commenced. Echocardiogram revealed evolving hypertrophic cardiomyopathy with asymmetrical interventricular septal hypertrophy that was treated with Propranolol. An umbilical hernia was surgically repaired. Ophthalmologic assessment showed mild left divergent squint but was normal otherwise. Audiology revealed bilateral glue ear at 16 months, which was treated with bilateral grommets. Despite a high calorie diet, growth remained slow with weight consistently between 0.4th and 2nd centiles; developmental milestones were appropriate for corrected gestational age.

At review aged 5.5years, patient 2 is developmentally appropriate, but both height and weight remain on 2nd percentile for age. She continues to take Riboflavin (10mg/kg/day), Coenzyme Q10 (5mg/kg/day) and vitamin C (100mg/day). Blood lactate remains slightly elevated at 2.49mmol/L and plasma amino acids show a slight increase in alanine reflecting raised lactate. Cardiology review at this time showed an overall improvement with a structurally normal heart with a single area of left ventricular thickening on midpoint of interventricular septum. Propranolol was discontinued in light of the normal cardiac function. She has recently undergone nasal cautery for recurrent nose bleeds and repeat grommet insertion, there were no problems with general anaesthesia although fasting time was minimised and IV dextrose 5% with saline was administered. Cutis marmorata on all limbs, a thin philtrum, prominent forehead and petite facial features were noted on physical examination.

There was no family history of note; her twin sister is well with height and weight on the 75th centile for age. Although parents are unrelated they originate from the same rural area in Ireland.

Patient 3

This female was born at 34+5/40 weeks by emergency LSCS, due to intrauterine growth retardation and oligohydramnios, with a birth weight of 1580g. Intravenous steroids (dexamethasone) were administered to mother prior to delivery to prevent respiratory distress in the neonate. No resuscitation was required at birth, APGARS were 9 at 1 minute and 10 at 5 minutes. She was admitted to the NICU for monitoring and developed respiratory distress requiring continuous positive airway pressure and she was treated with intravenous antibiotics. Blood lactates were persistently high (2-5mmol/L) though ammonia was normal. Echocardiogram on day 1 of life showed a large patent ductus arteriosus (which closed spontaneously), patent foramen ovale and tricuspid regurgitation.

Laboratory investigations included normal karyotype, negative TORCH screen and cranial ultrasound showed increased echogenicity in the periventricular white matter. Metabolic screens showed normal plasma amino acids whilst urinary organic acids showed a persistent, marked increase in lactate, fumarate and malate excretion. Acylcarnitine profile showed slightly decreased free carnitines (12.3μmol/L, normal range: 22-52μmol/L). She was discharged from the neonatal unit aged 6 weeks. At four months she had poor weight gain; growing below the 0.4th percentile and was hypotonic with mild motor delay. She had raised blood lactate (6.1–6.4mmol/L) and CSF lactate (3.5mmol/L) with an elevated lactate:pyruvate ratio. Fibroblast beta-oxidation of fatty acids was normal. Mild concentric left ventricular hypertrophy was noted on echocardiograph and abdominal ultrasound scan revealed hepatomegaly. Brain MRI at aged 4 months showed a mild delay in myelination but was otherwise normal. MRSpectroscopy (voxel over left basal ganglia) was also normal. A muscle biopsy was taken at age 8 months showed normal morphology and normal histochemical oxidative mitochondrial enzyme activities. Biochemical investigation of mitochondrial respiratory chain complexes revealed a marked deficiency of complex I relative to citrate synthase.

At review aged ten months, she was noted to have a prominent forehead, thin philtrum and petite facial features. Ophthalmology review was normal. Co-enzyme Q10 supplementation commenced (5mg/kg/day) She was admitted to a local hospital aged 23 months with adenoviral gastroenteritis; she was ketotic with metabolic acidosis and responded to intravenous fluids. Transoesophageal echocardiogram at 2 years excluded a ventricular septal defect and confirmed biventricular hypertrophy. Riboflavin supplementation was commenced aged 24months and she continues on this at age 30months (10mg/kg/day) together with co-enzyme Q10 (5mg/kg/day). She is developmentally appropriate for her age. Her weight is consistently below 0.4th percentile whilst her height is on 0.4th-2nd percentile. Systemic examination was normal. Review of blood results at 23months showed elevated lactate (3.7mmol/L), Cardiology review at 3years showed very mild left biventricular hypertrophy with normal left ventricular function.

Patient 3 is of Irish descent, has no siblings and there is no history of consanguinity or metabolic disease within the family.

Patient 4

Patient 4 is the second female child of unrelated Irish parents, born at 39 weeks gestation by elective LSCS. She was vigorous at birth with a birthweight of 2.32kg (0.4th-2nd centile) and head circumference of 31.4cm (2nd centile). She was admitted to ICU with jaundice due to ABO incompatibility and was noted to be hyponatraemic; this resolved prior to discharge at 8 days of age. She was also noted to have mild dysmorphic features including a large anterior fontanelle, a

prominent maxilla and a long philtrum. A skeletal survey showed no evidence of skeletal dysplasia. Weight gain and linear growth were both slow. Examination revealed a murmur and echo confirmed mild branch pulmonary artery stenosis. Cranial and renal ultrasound were normal. DiGeorge and Williams syndrome were excluded genetically and karyotype/microarray investigations were normal. In infancy, she was referred to regional endocrinology services for investigation of short stature and poor growth velocity. She had normal IGF-1 and IGF-BP3 levels (IGF-1 7.7nmol/L, IGFBP-3 1.7mg/L). She also had a normal glucagon stimulation test at age 3.5 years, with growth hormone >10 ng/mL at 90 minutes.

She presented to A&E at the age of 3 years 8 months following a vomiting illness with encephalopathy, seizures, high anion gap metabolic acidosis, raised serum lactate (3.34mmol/L), ketotic hypoglycaemia and hyperammonaemia (376µmol/L). Urine organic acids showed very marked ketonuria with very marked secondary dicarboxylic aciduria and hydroxydicarboxylic aciduria. A fasting study revealed persistently elevated lactates with normoglycaemia.

Brain CT was normal whilst cranial MRI revealed high signal in bilateral periventricular white matter and dentate nuclei, suggestive of a metabolic disorder. A muscle biopsy was taken which showed respiratory chain enzyme activities within the normal range when controlled for citrate synthase activity. Full mitochondrial DNA (mtDNA) sequencing investigations revealed no abnormalities. The patient attends the metabolic clinic for regular follow-up visits. She also attends the endocrinology clinic and shows poor catch up growth despite growth hormone therapy. She could be discharged from cardiology. She is of good general health and developmentally appropriate with normal vision, but mild conductive hearing loss. Current medications include co-enzyme Q10 5mg/kg/day and recombinant human growth hormone 30mcg /kg/day. There is no family history of note.

Patient 5

Patient 5 is the third male child of distantly-related parents. He was born at 37 weeks gestation by elective LSCS following symmetrical IUGR from 32/40 and unsatisfactory uteroplacental blood flow assessment. He had a birth weight of 2.02kg (0.4th-2nd centile), head circumference 31cm (2nd-9th centile), and length 45cm (9th centile). Apgars were 9 at 1 min and 9 at 5 min after delivery. He was admitted to SCBU with poor feeding. Thyroid-stimulating hormone (TSH) was found to be elevated on his Guthrie card and the diagnosis of congenital hypothyroidism was confirmed by thyroid function tests on day 7 (TSH 115, fT4 9.4). He had no episodes of hypoglycaemia and had a normal cranial USS. His thyroid scan was consistent with dyshormonogenesis. There is a paternal family history of renal problems, pigmentary retinopathy and hypothyroidism and his father had undergone subtotal thyroidectomy for hyperthyroidism.

He was admitted to his regional hospital at the age of 5 months for investigations for failure to thrive. The results of his liver function tests, full blood count, venous blood gases, serum amyloid A and toxoplasmosis, rubella, cytomegalovirus, herpes simplex, and HIV (TORCH) screen were all normal. Blood lactate was persistently elevated (up to 4.0mmol/L).and he was subsequently noted to have recurrent lactic acidosis when unwell. Urinary organic acids showed moderate ketonuria, elevated 3-methylglutaconate and 2-ethylhydracryllate excretion and TCA cycle intermediates. MRI and MRS was normal. Thyroxine, Co-Enzyme Q10, vitamin C and vitamin E were administered from age 20 months, (thyroxine was weaned at 3 years of age) with carnitine supplementation from 4 years of age which resulted in some weight gain.

Currently age 11 yr, his thyroid function, echocardiogram and ophthalmological assessment all remain normal. He has ongoing difficulties with concentration and coordination and has additional support in mainstream school. He had attended speech and language therapy until age 10 yr due to pronunciation difficulties, but has now been discharged. He continues to be an active child with good appetite and energy, but does have ongoing issues with poor weight gain and short stature. He remains on Co-enzyme Q10 (2.6mg/kg/day), carnitine (13mg/kg/day), Vitamin C (22mg/kg/day) and vitamin E (4.4mg/kg/day). He continues to attend regional endocrinology services following referral at 9 yr for short stature. His current height is 125.8cm (0.4th centile) and his weight is 22.75kg (0.4th centile). Growth velocity is 1.8cm/year. Mid parental height is on the 75th centile. Baseline bloods include IGF-1 12.9nmol/L (RR 10.8 to 63.7) and IGFBP-3 4.1mg/L (RR 1.6 to 5.0). Bone age correlates with chronological age. He has not received growth hormone treatment in accordance with parental wishes.

Patient 6

The parents of Patients 6 and 7 are unrelated and of Irish descent. They lost their first child on day 2 of life. She had been born at term by emergency LSCS for fetal distress. Birth weight was 2.8kg. She had an acute collapse on day 2 of life with a high anion gap metabolic acidosis and markedly elevated lactate (28mmol/L). Echocardiogram was consistent with persistent pulmonary hypertension of the newborn. Despite intensive care management with full inotropic support her condition deteriorated further with the development of multiorgan failure. Life support was withdrawn on day 2 of life. An underlying metabolic aetiology was suspected.

Patient 6 is a female born by elective LSCS at 37+4 weeks for oligohydramnios and IUGR with a birth weight of 1.96kg (<0.4th centile), length 44cm (<0.4th centile), and head circumference 32cm (2nd centile). Apgar scores were 9 at 1 min and 10 at 5 min after delivery. Given the family history of a female sibling dying on day 2 of life, she was electively admitted to SCBU for observation. On admission to SCBU blood glucose was recorded as 1.9 mmol/L and an intravenous infusion of 10%

dextrose corrected her blood glucose levels. She initially breastfed well but by day 3 was feeding poorly and had an elevated serum lactate level of 9.67mmol/L (normal range 0.7-2.1 mmol/L). She was recommenced on IV fluids and lactate levels improved. Amino acids were normal, however urine organic acids showed markedly raised lactate and ketonuria with minimal DCA and OH-DCA. Urinary 4 hydroxy-phenyllactate was also noted to be moderately elevated in keeping with mitochondrial dysfunction, liver dysfunction or prematurity. Feeding was recommenced and lactate levels improved. She was discharged aged 13 days and remained well at home, continuing to feed and thrive albeit slowly. Clinical review at 5 weeks of age showed normal brain MRI/MRS and normal echocardiogram.

Currently 2.5 yr she remains small (0.4^{th} - 2nd centiles for weight, 2nd -9th centiles for height, head circumference on the 25th centile), but continues to make developmental progress with age-appropriate skills and walked aged 15 months. She has mild facial dysmorphism with a prominent forehead, anisocoria, narrow philtrum and petite features. She receives physiotherapy, occupational therapy and speech and language therapy and CoQ10 supplementation (5mg/kg/day).

Patient 7

Patient 7 is the younger brother of patient 6. He was delivered by emergency LSCS at 36+5 weeks for IUGR and poor biophysical profile. He weighed 2.07kg (2nd-9th centile) with apgar scores of 9 at 1 min and 10 at 5 min. Similar to his sisters, he had elevated serum lactate levels (4.22-5.53mmol/L) and moderate increase in 4-hydroxy-phenyllactate on day 3 of life. Echocardiogram and cranial ultrasound were normal, as were ammonia, venous blood gases, renal and liver profiles. Physical examination revealed coronal hypospadias, left undescended testis and right inguinal hernia. He was petite and physically similar to his older sister. Currently age 8 months, he is making good developmental progress, but remains of small weight (<0.4^{th} centile), length (9th centile) and head circumference (2nd centile). He takes CoQ10 at 5mg/kg/day. He has persistent mildly elevated lactates (3-5mmol/L) and urinary organic acids show moderate increase in TCA metabolites, particularly fumarate. There is no evidence of ketosis although acylcarnitines show persistent slight elevation of hydroxybutyryl carnitine.

Patient 8

This male is the 4th child of non-consanguineous Caucasian parents of Irish descent; three older siblings are healthy and have no growth or metabolic problems. He was born at term by normal vaginal delivery weighing 2.56 kg. There was a history of poor feeding in early infancy, but development was normal and there were no hospital admissions or recurrent illnesses. He was referred to his regional paediatric endocrinology service because of poor growth at the age of 2.4 years. At this

time height was 80 cm (-3.1 SD) with weight of 9.6 kg (-3.1 SD). Parental heights were normal at 181 cm for father (+ 0.5 SD) and 153 cm for mother (-1.7 SD). Some mild facial dysmorphism, with relative macrocephaly, frontal bossing and deep-set eyes, was noted. Baseline investigations including FBC, U&E, LFT, coeliac screen and thyroid function tests were normal. He continued to grow poorly and a glucagon stimulation test was performed at the age of 3 years which demonstrated normal peak concentrations of growth hormone at 10.4 µg/L and cortisol at 640 nmol/L. Baseline IGF-I concentrations were undetectable (NR 4 -20 nmol/L), which along with the normal peak GH could indicate GH resistance. An IGF-I generation test, however, demonstrated a significant rise in concentrations of IGF-I (<3.2 nmol/L to 6 nmol/L) and IGFBP-3 (1.1 to 3 mg/L, NR 0.5 – 2.9 mg/L)) after 4 days of growth hormone at 50 µg/kg/day.

At the age of 3.4 years, he was commenced on recombinant human growth hormone therapy 0.5 mg (45 µg/kg/day) when his height was 83 cm (-4 SD). There was an initial period of catch up growth until 4.5 years when height reached 96 cm (-2.3 SD). He has continued on growth hormone and when last reviewed at age 9.1 years he was 121 cm tall (-2.2 SD) and weighed 21.5 kg (-2.2 SD).

Karyotype was 46 XY. Investigations for Silver-Russell syndrome were normal. Sanger sequencing of the *CUL7, OBSL1* and *CCDC8* genes - based on a presumed diagnosis of 3-M syndrome - did not identify any pathogenic variants. Brain MRI at the age of 8 years identified a small focus of high T2-weighted signal in the right external globus pallidus which was also evident on FLAIR imaging, but was otherwise normal. ECG showed mild tricuspid regurgitation but normal ventricular function. ECG revealed delta waves consistent with Wolff-Parkinson White syndrome, but there was no history of palpitations or admission for supraventricular tachycardia. Serum lactate level was normal (1.8 mmol/L; normal <2.2mmol/l).

Patient 9

Patient 9 is the younger female sibling of patient 8. She was born at term weighing 2.27 kg (<0.4th centile). At 3 years of age her height was 82.7 cm (<0.4th centile) and weight was 9.6 kg (<0.4th centile). She had facial dysmorphism with frontal bossing and deep-set eyes, similar to her brother. Baseline investigations including full blood count, liver function, bone profile, thyroid function, coeliac screen and karyotype were all normal. A glucagon stimulation test demonstrated a low peak GH concentration of 3.7 µg/L but normal cortisol concentration of 585 nmol/L. Baseline IGF-I and IGFBP-3 were within normal range and pituitary MRI was normal. Patient 9 had a moderate response to growth hormone therapy, at commencement aged 3.5 years her height was -3.2 SD and is now -2.4 SD at age 7 years. Echocardiogram and ECG remain normal and MRI brain at age 7 years was normal except for increased pituitary size. Blood lactate remains normal at 0.6 mmol/L (normal <2.2mmol/l).

Patient 10

This male is the first child of unrelated Northern Irish parents. He was born at 31 weeks gestation by elective LSCS due to severe IUGR and absent end-diastolic flow on uteroplacental blood flow assessment. Birth weight was extremely low at 730 g. He required intubation and ventilation on 3 occasions during the first 3 weeks of life due to lower respiratory tract infections and went on to develop chronic lung disease requiring several months of CPAP and supplementary oxygen. Despite a high calorie diet growth remained slow and his weight was consistently below the 0.4th centile. He was discharged from neonatal intensive care at 5 months, off all respiratory support, but requiring nasogastric feeds. He was noted to have hepatosplenomegaly, right inguinal hernia, hypospadias and mild developmental delay. MRI scan at 4 months showed partial agenesis of his corpus callosum.

He was admitted a few weeks later with rhinovirus bronchiolitis and required intubation and artificial ventilation. During this admission he was noted to be dysmorphic with overriding toes, single palmar creases, unusual eyebrows, and dysplastic ears. Echocardiogram revealed severe pulmonary hypertension with a dilated right heart, moderate right ventricular hypertrophy and dilated pulmonary artery. He received inotropic support, milrinone and nitric oxide. Blood lactate was elevated at 10 mmol/l with a normal pH of 7.41 and bicarbonate of 26 mmol/L. He had 3 episodes of necrotizing enterocolitis (NEC), successfully managed with conservative treatment and total parenteral nutrition (TPN), but required a laparotomy for a stricture in the descending colon at 6½ months of age. He remained ventilated for 11 weeks and required nitric oxide and sildenafil treatment for pulmonary hypertension for 12 weeks. By 8 months his pulmonary hypertension had resolved and he was off cardiac medication. His weight gain was very slow and he remained dependent on parenteral nutrition until 9 months of age. Review at 10½ months, (8 months corrected), shows him to be making good developmental progress with normal muscle tone. Ophthalmological assessment revealed a left non-paralytic convergent squint. He is fed via a nasogastric tube and but weight gain remains slow at 3.8 kg. He is on riboflavin (50mg/day) and thiamine (25mg /day); therapeutic ubiquinone was discontinued for intolerance (vomiting).

Patient 10 has been extensively investigated from a metabolic perspective with normal CK, lysosomal enzymes, long chain fatty acids, transferrin isoforms and sterols were all normal. A CGH microarray is normal. Skeletal survey shows no skeletal abnormalities. Urinary organic acids, performed while unwell, showed elevated lactic acid with large amounts of ketones 2 and 3 hydroxybutyrate with significantly elevated fumaric and malic acid. 4-hydroxy-phenyllactate was also elevated. Urinary organic acids show elevated lactate and TCA intermediates. Acylcarnitine and plasma amino acids were normal (with normal alanine 449 and proline 308). Skin biopsy showed abnormal flux studies, suggestive of primary mitochondrial disorder, but no evidence of a fatty acid oxidation defect.

Pyruvate dehydrogenase activity measured in these skin fibroblasts was low (0.36 nmol/mg/protein/min; normal range 0.6-0.9 nmol/mg/protein/min) however *PDHA1* sequencing was normal. Local muscle biopsy analysis revealed increased lipid deposition, though he was receiving TPN at the time of the biopsy.
