# Supplementary material for: A recurrent mitochondrial p.Trp22Arg NDUFB3 variant causes a distinctive facial appearance, short stature and a mild biochemical and clinical phenotype
Source: J Med Genet. 2016 Apr 18;53(9):634–41. doi: 10.1136/jmedgenet-2015-103576 (PMC5013090; doi:10.1136/jmedgenet-2015-103576)
Supplement: Supplementary table [file jmedgenet-2015-103576supp_table.pdf]

Supplementary Table 1. Genes included on a custom Ampliseq "Isolated complex I deficiency" target capture and their corresponding GenBank reference sequences.

	Gene	Reference sequence
1	*NDUFA1*	NM_004541.3
2	*NDUFA2*	NM_002488.4
3	*NDUFA3*	NM_004542.3
4	*NDUFA5*	NM_005000.4
5	*NDUFA6*	NM_002490.3
6	*NDUFA7*	NM_005001.3
7	*NDUFA8*	NM_014222.2
8	*NDUFA9*	NM_005002.4
9	*NDUFA10*	NM_004544.3
10	*NDUFA11*	NM_175614.4
11	*NDUFA12*	NM_018838.4
12	*NDUFA13*	NM_015965.6
13	*NDUFAB1*	NM_005003.2
14	*NDUFB1*	NM_004545.3
15	*NDUFB2*	NM_004546.2
16	*NDUFB3*	NM_002491.2
17	*NDUFB4*	NM_004547.5
18	*NDUFB5*	NM_002492.4
19	*NDUFB6*	NM_002493.4
20	*NDUFB7*	NM_004146.5
21	*NDUFB8*	NM_005004.2
22	*NDUFB9*	NM_005005.2
23	*NDUFB10*	NM_004548.2
24	*NDUFB11*	NM_019056.5
25	*NDUFC1*	NM_002494.3
26	*NDUFC2*	NM_004549.5
27	*NDUFS1*	NM_005006.6
28	*NDUFS2*	NM_004550.4
29	*NDUFS3*	NM_004551.2
30	*NDUFS4*	NM_002495.2
31	*NDUFS5*	NM_004552.2
32	*NDUFS6*	NM_004553.4
33	*NDUFS7*	NM_024407.4
34	*NDUFS8*	NM_002496.3
35	*NDUFV1*	NM_007103.3
36	*NDUFV2*	NM_021074.4
37	*NDUFV3*	NM_001001503.1
38	*NDUFAF1*	NM_016013.3
39	*NDUFAF2*	NM_174889.4
40	*NDUFAF3*	NM_199069.1
41	*NDUFAF4*	NM_014165.3
42	*NDUFAF5*	NM_024120.4
43	*NDUFAF6*	NM_152416.3
44	*NDUFAF7*	NM_144736.4
45	*ACAD9*	NM_014049.4
46	*NUBPL*	NM_025152.2
47	*FOXRED1*	NM_017547.3
48	*ECSIT*	NM_016581.4
49	*TMEM126B*	NM_018480.3
