# Supplementary material for: A recurrent mitochondrial p.Trp22Arg NDUFB3 variant causes a distinctive facial appearance, short stature and a mild biochemical and clinical phenotype
Source: J Med Genet. 2016 Apr 18;53(9):634–41. doi: 10.1136/jmedgenet-2015-103576 (PMC5013090; doi:10.1136/jmedgenet-2015-103576)

Supplementary Figure. Haplotype analysis to investigate a putative founder mutation.

Genotyping results for markers D2S309, D2S2214, D2S116 and D2S2309 are shown for Patients 1-10 as well as the two previously reported cases documented in the literature (denoted RC1[10] and RC2[9]).

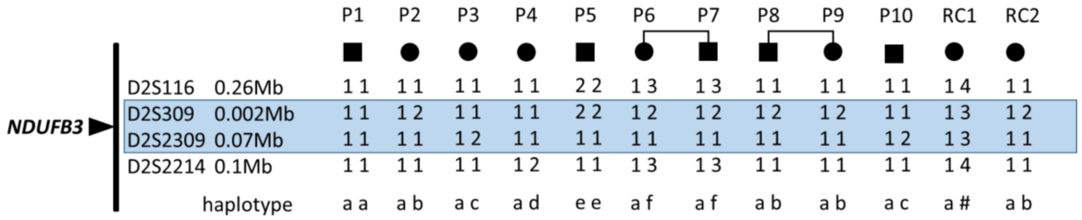

Supplement: Supplementary figure [file jmedgenet-2015-103576supp_figure.pdf]
